# Macromolecular crowding alters transcription: real-time measurements with SYBR Green II

**DOI:** 10.21203/rs.3.rs-8854472/v1

**Published:** 2026-03-24

**Authors:** Suleyman Ucuncuoglu, Md Abul Kalam Azad, Narendar Kolimi, Laura Finzi, David Dunlap

**Affiliations:** Clemson University; Clemson University; Clemson University; Clemson University; Clemson University

## Abstract

Various factors affect transcription, including regulatory proteins, promoter sequences, ionic conditions, and nucleotide concentrations. Gel electrophoresis of radiolabeled RNA is a widely used assay to quantify transcript production in vitro. However, the use of radioactive reagents requires hazardous chemical training as well as specialized protocols and imaging systems. Moreover, dynamic monitoring of in vitro transcription could provide insight into conditions affecting the reaction. Widely available microplate readers and quantitative PCR instruments could be broadly adopted to study the kinetics and yields of transcription reactions with a suitable fluorescence assay. The SYBR Green II fluorophore has high affinity for RNA and can be used to monitor RNA production from *in vitro* transcription assays. In experiments described herein, the effect of macromolecular crowding agents on transcription by *Escherichia coli* RNA polymerase was characterized. Polyethylene glycol (PEG) 2000 inhibited transcription more than PEG 8000 which displayed re-entrant behavior as a function of concentration. Glycerol had little effect and Dextran 70 stimulated transcription by approximately 20%.

## INTRODUCTION

With the discovery of the regulatory roles of RNA molecules and their use in RNA therapeutics, demand for RNA synthesis is increasing (1,2). Therefore, a rapid, fluorescence-based screening method with which to assess transcription would facilitate efforts in the biotech and pharmaceutical industries as well as in biological and biomedical research. Quantifying in vitro transcriptional activity is critical for understanding the effects of various parameters, such as different promoter sequences, reaction conditions (3), reaction components (4), and the relative kinetics of wild-type or mutant RNA polymerases from different organisms (5). Ideally, such quantification would include dynamic measurement of the RNA produced. The incorporation of radioactive nucleotides into nascent RNA during transcription is a trusted technique for observing transcription in various conditions (6,7). Although radioactive labeling offers high detection sensitivity, the regulations for handling radioactive reagents and samples, the cost, and the need for specialized training and equipment limit use of the technology.

Measuring the amount of RNA produced using micro volume spectrophotometers could be used to quantitatively assess the production of RNA. Measuring RNA production at various time points in a reaction would produce a dynamic profile of transcription, but it is quite laborious without automated liquid handling. In addition, such measurements are most accurate after purification of the RNA, introducing a step in which the product to be assayed may be incompletely recovered (8).

Technological advances in fluorescent probes and their detection have significantly enhanced biotechnological methods for investigating biological systems (9,10). For example, a real-time, quantitative polymerase chain reaction (PCR) that relies on Fluorescein amidite (FAM) or SYBR Green I dyes (11) is a powerful tool with which to quantify DNA samples. Fluorescence could be exploited to produce a detectable readout of transcription utilizing fluorophores that bind to RNA aptamers (12). In this case, the template must include a sequence encoding an RNA aptamer such as Broccoli, which adds additional complexity. Instead, by employing a fluorescent dye, such as SYBR Green II, that preferentially binds to RNA (13), transcriptional activity can be monitored without sequence manipulation or radioactive RNA labeling, using widely available equipment such as microplate readers and real-time PCR (RT-PCR) instruments. Real-time monitoring of such fluorescence measurements also conveniently produces information on transcription kinetics. Furthermore, SYBR Green II is cost-effective, especially compared to radioactivity-based detection methods. This makes SYBR Green II-based transcription assays particularly attractive for monitoring in vitro transcription reactions.

To validate this method, we monitored the transcriptional output of *E. coli* RNA polymerase. Using optimized reaction conditions, we examined the inhibition of transcription by molecular crowders, which are used to mimic the crowded intracellular environment.

## MATERIAL AND METHODS

The Fluc plasmid is available in the HiScribe T7 High Yield RNA Synthesis Kit (NEB, New England Biolabs, Ipswitch, MA). The pDM_N1_400 plasmid (5848 bp) was described by Xu et al. (14). It contains a T7A1 promoter and a lambda t1 terminator with a transcript length of 1279 nucleotides, Fig. S2. Biotinylated RNA polymerase was prepared as described(15). RiboRuler High Range RNA ladder (Thermo Fisher Scientific, Waltham, MA), was used as a gel electrophoresis standard.

### Transcription Reactions

To study how macromolecular crowding affects transcription, we carried out in vitro transcription reactions containing different types and concentrations of macromolecular crowders. All reactions were 20 μL volumes prepared in 8-strip qPCR tubes (TCS0803/TLS0801, Bio-Rad, Hercules, CA) and were kept on ice until measurement.

Transcription reactions were prepared in 20 μL volumes containing 40 mM Tris-HCl, 150 mM KCl, 10 mM MgCl2, 1 mM DTT, 0.01% Triton X-100, pH 7.5, 2 mM total NTP (NEB, Lot: 10300726), and 2.5X SYBR Green II (Cat. No. 12510, Lumiprobe, Westminster, MD). A master mix was used to minimize pipetting and maximize uniformity between reactions. As an alternative to SYBR Green II, a fluorophore with high specificity for binding RNA, Quantifluor (Promega, Madison, WI), was also tested at dilutions indicated by the manufacturer.

After aliquoting 6.83 μL of master mix per reaction, 2 μl of E. coli RNA polymerase holoenzyme (NEB) and desired volumes of macromolecular crowder were added. The final reaction volume was adjusted to 20 μL using nuclease-free water. Positive control reactions contained no crowder, while experimental reactions included various macromolecular crowding agents at different weight/volume fractions. The negative controls had no RNAP or crowders. All samples were mixed gently on ice and immediately transferred to the qPCR instrument for measurement.

Fluorescence data were collected using the SYBR channel of a CFX Connect Real-Time PCR Detection System (Bio-Rad). Reactions started upon warming from 4 to 37°C, and fluorescence intensity was recorded at 1 minute and then every 10 minutes thereafter for 180 minutes. Each measurement lasted about 2–3 seconds. Baseline subtraction was disabled in the CFX Maestro software so that raw fluorescence values could be analyzed directly.

Each condition with associated negative and positive controls was repeated at least three times (Figs. S3 and 4). Time courses of fluorescence intensities were compared across crowder types and concentrations to assess how macromolecular crowding influenced transcription.

The fluorescence intensities varied considerably between experiments performed on different days. Therefore, a strategy for normalizing fluorescence intensities similar to that previously published was adopted (13). Briefly, all time courses of intensities were offset to begin at zero. Then, the values of the negative control sample were subtracted from the other time courses. Finally, the fluorescence intensities were normalized by dividing by the maximum intensity for the positive control. Confidence intervals of the mean fluorescence versus time profiles were calculated by multiplying the appropriate two-tailed inverse value from Student’s t distribution and standard error of the mean values:

$$\text{CI}=tinv\left(\text{\%},\text{N}\right)\text{*SEM};\;\text{interval}=\text{mean}\pm \text{Cl}$$

Negative controls displayed decreasing fluorescence versus time, which has been documented previously and corrected for as described (13). Data were discarded from final analysis if normalized fluorescence values were negative, fluorescence peaked and decreased during the experiment, or values were extreme outliers. Graphs were produced using Matlab (Mathworks, Natick, MA).

### Nanodrop measurements

20 μL of RNase-free water, 5 μL DNA Digestion buffer, and 5 μL DNase-I were added to the transcription reactions to eliminate the template DNA. The mixture was incubated at 25°C for 15 minutes. The remaining RNA was purified as per instructions for an RNA Clean & Concentrator - 5 kit protocol (R1014, Zymo Research, Tustin, CA). A Nanodrop Lite device (Thermo Fisher Scientific, Waltham, MA) was used to measure the concentration of purified RNA.

## RESULTS

*In vitro* transcription reactions generally combine an RNA polymerase, nucleotide triphosphates, and a DNA template containing a sequence with a promoter, gene body, and terminator in a buffer including magnesium. An efficient promoter from bacteriophage T7 is often used for in vitro transcription reactions in industry and academic research (16). Appropriate concentrations of nucleotides and buffer conditions were selected from trials with two different RNA polymerases. One was T7 RNA polymerase, a widely used, single subunit RNA polymerase (2). Another was holoenzyme *E. coli* RNA polymerase (RNAP) which has five core subunits and efficiently recognizes promoter sequences when reconstituted with a sigma subunit (17–19). Distinct bands of RNA were produced in vitro using a biotinlylated *E. coli* RNA polymerase on 500 ng aliquots of two different templates to show proper initiation and termination of transcription (Figure S1). These reactions were incubated for 150 minutes at 37 degrees before treating aliquots with DNase I, isolating RNA with a cleanup kit, spectrophotometrically measuring the RNA concentration, and visualizing the RNA transcripts by electrophoresis. For these measurements, transcription that initiated from the bacteriophage T7A1 promoter and terminated at the Lambda T1 terminator (Fig. S2) was first verified with spectrophotometric measurements (Table 1). Using large amounts of template DNA with the highly specific, processive T7 RNA polymerase produced large amounts of RNA. *E. coli* RNA polymerase produced smaller amounts of RNA from smaller amounts of template DNA especially with linearized template. Reactions containing Quantifluor, a fluorescent molecule advertised to very specifically bind RNA, or SYBR Green II, a fluorescent molecule that binds both DNA and RNA produced similar amounts of transcript.

To develop a straightforward, multiplexed protocol for monitoring transcription with fluorescence, the amount of template DNA was optimized. Too much template DNA might produce high background if the supposedly highly specific fluorophore were to bind DNA as well as RNA. Too little DNA template would fail to produce enough transcript and associated fluorescence. An ideal amount of template DNA would maximize the production of RNA without generating too much fluorescence. The fluorescence intensities of 2.5X SYBR Green II with different amounts of template DNA were measured with a qPCR instrument (Figure S3). During measurements lasting 3 hours, the fluorescence intensity traces for each sample were stable and directly correlated with the amount of DNA template. One or ten ng of DNA in a 20 ul reaction volume produced indistinguishable, low levels of fluorescence. Instead, 100 or 500 ng of template increased fluorescence approximately 1.5- or 4-fold respectively. Further experiments were conducted with 100 ng of template DNA to maximize RNA production without excessive levels of fluorescence due to SYBR Green II staining of the template DNA.

Although reactions containing Quantifluor produced RNA, they displayed no steady increase of fluorescence during the time course of the incubation at 37 degrees (not shown) while SYBR Green II fluorescence rose steadily (Figure S4). The quantum yield of SYBR Green II is greater when bound to RNA than to DNA and much higher than that of ethidium bromide bound to RNA making it useful for standardization of RNA samples in hybridization studies (13). Only complete mixtures of the necessary components for transcription (DNA template, NTPs, RNA polymerase) and fluorescence (intercalating dye) exhibited significantly increasing fluorescence intensity.

One potential use of this fluorescence assay for transcription might be to assess the effects of inhibitors. Small molecules like rifampicin prevent nucleotide binding to the active site to strongly inhibit polymerization (20). However, even low concentrations of rifampicin dramatically quenched SYBR Green II fluorescence (not shown) as has been noted in other fluorescence studies (21,22). Therefore, the assay might be most suited to study RNA polymerase mutations or reaction conditions that alter transcription without introducing photoactive molecules.

One obvious condition is that transcription happens in a crowded cytoplasmic environment where proteins, nucleic acids, and metabolites can take up to 40% of the volume (23,24). Macromolecular crowding is known to alter the stability, conformations, and reactivities of proteins and DNA (25–31). Effects on individual steps of complex biochemical reactions like transcription initiation indicate surprising sensitivity of slight conformational changes to crowding agents (32). Biochemical reactions can be influenced by this macromolecular crowding in multiple ways. For example, crowding can promote complex formation and stabilize compact conformational states through excluded volume effects but also slow diffusion due to increased viscosity, especially at high crowder volume fractions. This might lead to differing effects on various steps of the transcription cycle, open complex formation, promoter escape, elongation, and termination with some enhanced and others inhibited (33). Experimental studies using synthetic crowders such as polyethylene glycol (PEG), Ficoll, Dextran, and glycerol have shown both inhibitory and stimulatory effects on transcription, often in a non-monotonic fashion (32). The SYBR Green II assay was an ideal method to assess the effect of macromolecular crowding on transcription. Therefore, we systematically used it to determine the impact of different crowder concentrations and polymer types on *in vitro* transcription.

### PEG 8000 inhibits transcription

**Figure 1:**
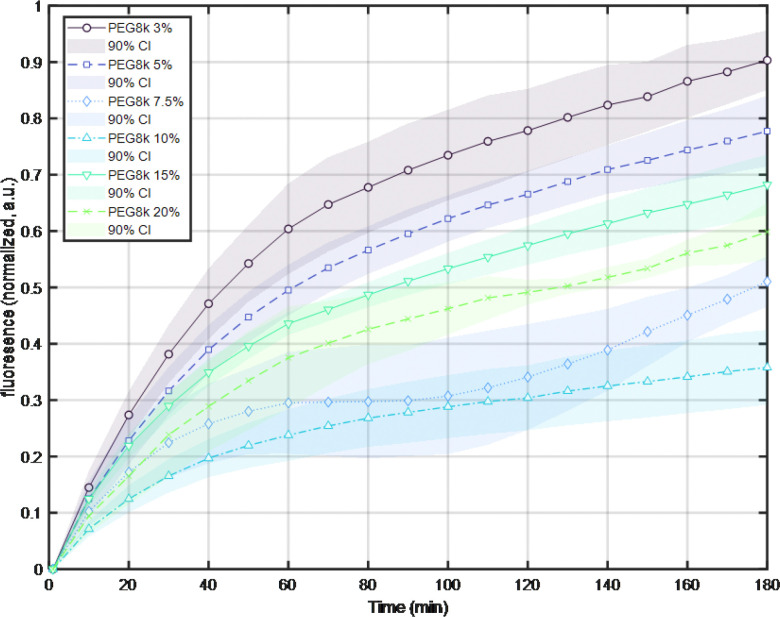
PEG inhibited transcription. The fluorescence intensities of mixtures of SYBR Green II, *E. coli* RNAP, NTPs, and plasmid DNA, in buffer with increasing amounts of PEG 8000 were monitored for 3 hours. Increasing the volume fraction of PEG from 3 to 10%, progressively inhibited average, normalized fluorescence intensity, (transcription). However, more PEG was less inhibitory. Each curve is an average of at least three replicates (see Fig. S5 for individual trials). Shaded areas indicate 95% confidence intervals of the mean.

High molecular weight polyethylene glycol is used pharmaceutically and in biophysics research to reconstitute an artificially “crowded” macromolecular environment. As the volume fraction of PEG increases, an initial trend in the behavior of an affected reaction develops but may reverse beyond a certain volume fraction as mentioned previously. This is apparent in Fig. 1 in which transcription in the presence of a low amount of PEG 8000 is slightly inhibited with respect to a reaction with no PEG 8000. As the volume fraction of PEG 8000 increased from 3 through 10%, transcription decreased from 90% to just below 40%. However, further increases in the volume fraction of PEG 8000 displayed inhibition of only 70 and 60% at 15 and 20% volume fractions respectively.

### PEG 2000 inhibits but Dextran 70 enhances transcription

**Figure 2:**
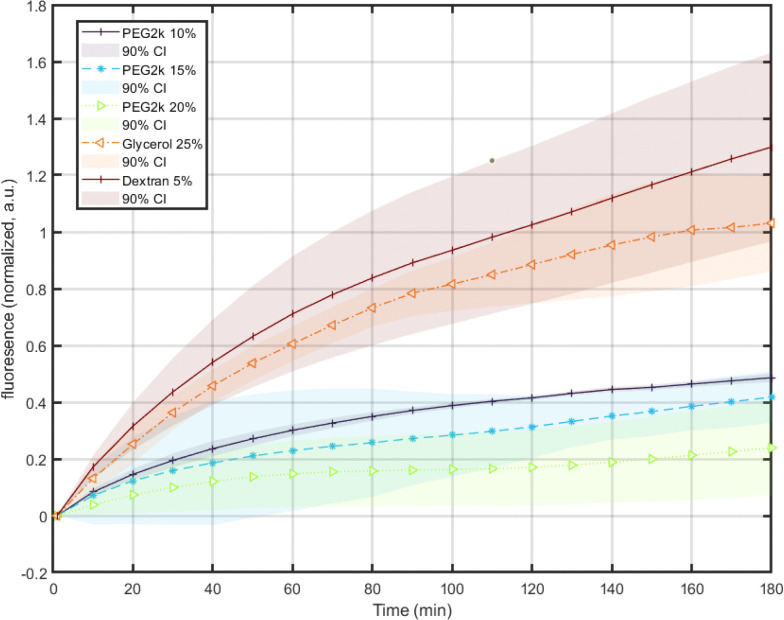
10, 15, or 20% volume fractions of PEG 2000 inhibited transcription almost as much as the most inhibitory volume fraction of higher molecular weight PEG 8000. Instead, the effect of 25% glycerol was negligible while 5% Dextran 70 enhanced transcription slightly. Each curve is an average of at least three replicates (see Fig. S6 for individual trials). Shaded areas indicate 95% confidence intervals of the mean.

PEG 2000 more strongly inhibited transcription at 10–20% volume fractions with no reversal evident at 15% (Fig. 2). This is consistent with the greater inhibitory efficacy of lower molecular weight PEG in a report in which all crowding agents and viscogens inhibited promoter escape (32). However, in a conflicting report PEG enhanced promoter escape while inhibiting the transition to an open promoter complex (33). While crowding may modify the kinetics of individual steps of transcription in different ways, even in a promoter dependent fashion, a key feature of the SYBR Green II measurements reported here is the determination of overall RNA output encompassing all three phases of transcription including initiation, elongation, and termination. In this assay, introducing a 25% volume fraction of glycerol had little effect while 5% Dextran 70 enhanced transcription. Glycerol is a small molecule that alters viscosity and is not expected to produce a significant excluded volume that would cause RNA polymerase or the DNA template to aggregate. It has been shown to inhibit initiation (32) however, it might also stabilize RNAP–DNA complexes to enhance elongation efficiency. Instead, Dextran 70 is larger and could produce excluded volume around RNAP and DNA templates to drive them together and enhance transcription (34).

## DISCUSSION

Different methods for measuring transcriptional activity offer distinct advantages depending on the experimental needs. Radioisotope labeling remains the gold standard for RNA visualization, offering exceptional sensitivity with the ability to detect picogram amounts of radiolabeled RNA and distinguishing multiple RNA products generated in the same reaction, even at single-nucleotide resolution. However, the regulatory constraints, high costs, and hazards associated with handling and disposing of radioactive materials limit its use to specialized facilities with proper radiation safety infrastructure. Moreover, in many cases, a high yield of RNA is the primary objective, which does not require the sensitivity of radioisotope labeling. Nanodrop spectrophotometry can be ideal for RNA quantification due to the minimal sample volume required (0.5–2 μL) which conserves valuable reagents. Results are available within seconds, making the Nanodrop assay common worldwide, however, it is not multiplexed for high throughput.

In contrast, qPCR or plate reader instruments that can automatically read multi-well plate formats of transcription reactions spiked with SYBR Green II are attractive for high-throughput capability and high sensitivity. The method is particularly effective for monitoring transcriptional activity over time and readily displays the time beyond which RNA production plateaus. The method provides hands-free, quantitative results within ~ 2–3 hours, appropriate for absolute or relative transcriptional analyses using reference DNA templates. Like any assay, it requires optimization, but the versatility, cost-effectiveness, and dynamic output make it a competitive alternative to other methods.

Our results show that different crowding agents exert distinct effects on RNAP transcription efficiency. PEG 2000 reduced transcription relative to the control, with the strongest inhibition observed at the highest volume fraction, 20%. However, 15–20% PEG 8000 was less inhibitory than lower volume fractions. This suggests a balance between excluded volume enhancement that can concentrate reactants and interference with diffusion that can limit enzymatic reactions.

In contrast, 25% glycerol produced insignificant change in transcription. Glycerol is thought to promote hydration and might catalyze protein folding and reduce conformational fluctuations, potentially improving RNAP activity and elongation efficiency (35). These appear to be mild effects at low volume fractions (12), and may vary with different promoters (36).

On the other hand, 5% Dextran 70 enhanced transcription. Its large size likely creates excluded volume effects that increase the effective concentration of RNAP and NTPs, thereby boosting reaction rates.

Together, these findings emphasize that macromolecular crowding effects are agent-specific and size- and concentration-dependent. Importantly, the PEG results show that the relationship is not necessarily monotonic, underscoring the need to evaluate both chemical nature and amount of crowding agents when interpreting their effects on transcription.

The method used to assess *in vitro* transcription will likely depend on the specific goals of a study. For routine RNA quantification of a few samples, a Nanodrop is simple and efficient. Radioisotope labeling may be best for conditions requiring the highest sensitivity, if the proper facilities are available. For dynamic transcription analysis, SYBR Green II readout is a widely accessible, high-throughput, and sensitive measurement option. The assay might be particularly advantageous for screening chemical or natural product libraries for inhibitors of bacterial RNAPs to develop novel antibiotics if SYBR Green II quenching can be avoided. High-throughput monitoring of *in vitro* transcription in real time would be very useful in the development of high fidelity, highly processive mutants of RNA polymerases for RNA production.

## Supplementary Material

Supplementary Files

This is a list of supplementary files associated with this preprint. Click to download.

• floatimage1.png

• SUPPLEMENTARYINFORMATION.docx

SUPPLEMENTARY DATA

Supplementary Data are available at NAR online.

## Figures and Tables

**Table 1:** RNA produced in different reaction mixtures (red) was assayed spectrophotometrically. Both T7 and *E. coli* RNA polymerase (blue) were tested with two different templates (green) in two different buffers (brown).

buffer (NEB)	T7	T7	T7	T7	T7	*E. coli*	*E. coli*
polymerases (NEB)							
HiScribe T7 High yield RNA synthesis kit (diluted 10X)	0.1	0.1	-	-	-	-	-
*E. coli* RNA polymerase holoenzyme (diluted 10X)	-	-	0.1	0.1	0.1	0.1	0.1
templates							
Fluc DNA (NEB, ng/ul)	-	100*	-	5	5	-	5
pDM_N1_400 (ng/ul)*	100	-	5	-	-	5	-
fluorescent dye							
Quantifluor	-	-	+	+	-	+	+
SYBR Green II (5 ul of 10X)			-	-	+	-	-
RNA yield							
RNA produced (ng)	2060	1300	46	177	220	16.8	51.5

T7 RNA polymerase produced large amounts of RNA (darker red) with 100 nanograms of linear (*) DNA templates. *E. coli* RNA polymerase produced several micrograms of RNA from 5 nanograms of supercoiled Fluc plasmid templates and less from linear pDM_N1_400 template. Reactions that included Quantifluor or SYBR Green II produced similar amounts of RNA.

## Data Availability

The data underlying this article are available at https://figshare.com/s/a3d9323ad71ae5d75eec.
